# Disconnection Between Self-Reported Wellbeing and Heart Rate Variability from Wearables

**DOI:** 10.3390/s26041325

**Published:** 2026-02-19

**Authors:** Corey T. Ungaro, Anthony S. Wolfe, Zadok J. Isaacs, Peter John D. De Chavez, Eric C. Freese

**Affiliations:** 1 Gatorade Sports Science Institute, PepsiCo R&D, Chicago, IL 60607, USA; 2 Gatorade Sports Science Institute, PepsiCo R&D, Frisco, TX 75034, USAzadok.isaacs@pepsico.com (Z.J.I.); eric.freese@pepsico.com (E.C.F.); 3 PepsiCo R&D, Data Science and Analytics, Plano, TX 75024, USA; peter.dechavez@pepsico.com

**Keywords:** heart rate variability, wearables, wellbeing, health, activity tracker, readiness, recovery

## Abstract

Study Objective: To assess the association between daily self-reported emotional and mental readiness and their association with overnight resting heart rate variability. Methods: Participants included 21 males (32 ± 7 years) and 18 females (28 ± 4 years). Activity tracker data were collected for three months, measuring heart rate variability, resting heart rate, sleep time, sleep efficiency, bed/wake times, steps counted, and daily self-reported caffeine use, alcohol use, hydration, feelings on stress, illness, motivation, energy, nervousness, emotional stability, and recovery. Results: Self-reported stress and nervousness did not have an association with heart rate variability (Stressed HRV 63.5 ± 0.6 vs. Not Stressed HRV 63.1 ± 0.5 ms, *p* = 0.63) and (Nervous HRV 62.9 ± 0.9 vs. Not Nervous HRV 63.7 ± 0.4 ms, *p* = 0.41). Self-reported feelings of being energized had a negative association with heart rate variability (Energized HRV 64.1 ± 0.4 vs. Not Energized 66.9 ± 0.8 ms, *p* < 0.01). Conclusions: Subjective feelings of readiness may not correspond to activity tracker biometrics and should be taken into consideration when calculating readiness scores and providing personalized recommendations based on HRV. Caution should be taken when using HRV-based recommendations to guide a user’s health and wellness journey.

## 1. Introduction

Wearable activity trackers are ubiquitous across all ages, populations, and countries and have emerged as one of the most popular and widely implemented devices in the health and wellness industry. According to the International Data Corporation Worldwide Quarterly Wearable Device Tracker, the global wearables market reached 534.6 million units sold in 2024, reflecting a 5.4% year-over-year growth [1].

Wearable devices are typically equipped with two predominant sensors: photoplethysmography (PPG), an optical heart rate sensor, and tri-axial accelerometry, an acceleration sensor. When using these sensors, wearable devices can track a user’s steps, estimate energy expenditure, measure physical activity levels, sleep patterns, body temperature, oxygen saturation, heart rate, heart rate variability, and provide users guidance and education to improve or maintain their health and wellbeing.

One of the main attractions of wearable devices is their recovery and readiness scores, which are typically modeled using resting heart rate (RHR), heart rate variability (HRV), and sleep metrics to assess a user’s next-day readiness and provide users with feedback and recommendations [2]. These readiness scores are heavily influenced by HRV due to the greater degree of day-to-day variability and individually dependent metric compared to sleep and RHR measures [2,3,4,5]. Examples of some recommendations are, “your HRV lowered last night, you should take it easy today, or you had a great night of sleep, you are primed to tackle a new challenge today,” which may or may not reflect the users’ motivations [6].

HRV is a measure of cardiac vagal tone that can be quantified through the application of spectral analysis of the beat-to-beat (R-R) intervals [7], which are influenced by the balance between the sympathetic “fight or flight” and the parasympathetic “rest-and-digest” nervous system. Higher levels of HRV, especially in vagally mediated metrics like the root mean square of successive differences (RMSSD), largely reflect a more adaptable, resilient autonomic state. Lower HRV, by contrast, is associated with stress, fatigue, and diminished physiological flexibility [3,8]. HRV has been identified as a predictive factor for increased disease and cardiovascular mortality.

A qualitative examination on exerciser’s experiences using readiness/recovery scores to tailor their daily workouts showed that one of the main themes from user interviews was that “You can’t really capture complexities of a human on a device”. The authors state that there is growing recognition that physical, perceptual, cognitive, and affective states—which impact decisions to and experience of performing exercise—can fluctuate more frequently over time (hours, days) and across varying contexts within individuals. One subject was quoted saying, “I just noticed that the aches and things started to get to me, but it hadn’t changed the actual readiness or recovery score. It was more my own experience and my own perceptions of how I was feeling” [6].

Recent investigations into the association between HRV metrics from wearable devices and self-reported wellbeing have yielded inconsistent and sometimes confounding results. For example, research involving pre-school-aged children demonstrated that elevated levels of self-reported stress were associated with lower HRV [9]. Another study with military personnel revealed a modest correlation between HRV and perceived physical fitness, but no significant link was associated with perceived mental fitness [10]. The association between HRV and users’ subjective experiences of stress, nervousness, and energy remains underexplored. Given the widespread adoption of wearable devices and their role in guiding user behavior, further investigation into this connection is essential.

The primary aim of this study was to conduct an exploratory analysis of the association between HRV and daily self-reported wellbeing questions. The hypothesis was that negative emotional, mental, and physical wellbeing responses would correlate with low HRV measurements.

Significant and interesting findings were observed when comparing HRV and self-reported questions such as “Do you feel any stress?” and “Do you feel energized?”. To date, no study has investigated these comparisons of daily self-reported wellness and wearable biometrics over a 3-month period.

Knowing the popularity of wearable devices and how they influence and guide user behavior, it is of scientific importance to investigate the degree to which these personalized recommendations relate to how the individual feels and whether subjective metrics should be included in the evolution of personalized recommendations and feedback from wearable devices.

## 2. Materials and Methods

### 2.1. Study Inclusion and Exclusion Criteria

Subjects needed to be between the ages of 21 and 50 years old. Females must not have been pregnant, breastfeeding, or planning to become pregnant. All subjects needed to be able to read, write, and speak English and have no medical or other conditions that could interfere with informed consent, study protocol, or safety. Subjects needed to wear their wrist-worn activity device continuously, except when fully submerged in water. Subjects needed to understand all study procedures and were willing to sign the consent form.

### 2.2. Participants

Thirty-nine participants completed three months of real-world data collection with continuous monitoring using a wrist-worn activity tracker. Eleven subjects who did not meet the study requirements over the 3 months were excluded. Twenty-one males (age 32 ± 7 years; body mass index 27.5 ± 4.0 kg/m^2^) and eighteen females (age 28 ± 4 years; body mass index 25.4 ± 4.0 kg/m^2^). See Table 1 for descriptive statistics for each group. The study was approved by the Sterling Institutional Review Board (Atlanta, GA, USA) and funded by PepsiCo. Each participant gave written informed consent via DocuSign before starting the study.

### 2.3. Prospective Observational Longitudinal Study Design

After giving informed consent, subjects were instructed to complete a general health and demographics questionnaire via DocuSign. Following the completion of all questionnaires, subjects were provided with a Whoop 4.0 verison (Whoop, Boston, MA, USA) wrist-worn activity tracker and instructions on how to set up, charge, and sync their Whoop devices. Whoop calculates HRV using the root mean square of successive differences (RMSSD), a well-established and reliable indicator of parasympathetic nervous system activity [3,11,12]. HRV measurements represent the raw RMSSD nightly averages calculated at rest from Whoop. Sleep cycles of less than 4 h were omitted from the dataset. Subjects were instructed to wear their Whoop bands on their non-dominant hand continuously for three months, removing them only when necessary (i.e., charging, swimming, sport requirement).

### 2.4. Measurements

Whoop is a commercially available and validated wrist-worn three-axis accelerometer with an optical photoplethysmogram (PPG) sensor that tracks changes in motion patterns, altitude, location, and heart rate [11]. Objective measurements of mean HRV were generated during Whoop’s proprietary algorithm for automatic sleep and wake detection. Individual HRV baselines were established during the first 4 weeks of the study. Additionally, participants responded either “True” or “False” for the following daily wellbeing questions: felt nervous or anxious; had any alcoholic drinks; had caffeine; felt emotionally and mentally stable; felt energized; hydrated sufficiently; felt motivated; felt recovered; felt sick or ill; and felt stressed.

### 2.5. Data Analysis

All Whoop data were stored in the Gatorade Sports Science Institute’s electronic information management system. Whoop data was encrypted both at rest and in transit using AES 256-bit encryption. End users controlled their Whoop data and did not share it without consent. Only the subject’s pairwise data were included in the dataset. Any data that was not matched from the wearable’s biometric data and daily wellness survey questions were excluded from the final data analysis.

Statistical analyses were conducted using Minitab version 22.1 Statistical Software (Minitab, LLC, State College, PA, USA). De-identifiable IDs were used to determine and analyze the subjects’ raw data files. Informed consents were encrypted at transit and at rest and administered through DocuSign via email.

Analysis of variance (ANOVA) measured and determined the association between wellbeing entries “True” or “False” compared to HRV. Cohen’s d calculated the effect size when it was appropriate. Levene’s test assessed homogeneity of variance between groups. Normality of residuals was assessed using Kolmogorov–Smirnov or Shapiro–Wilk tests and graphically via frequency distributions and Q-Q plots. If departures from normality were found, data transformation was applied (e.g., logarithmic). Statistical significance was set at *p* < 0.05 for all descriptive tests. Data are presented as mean ± standard error.

## 3. Results

The primary aim of this study was to analyze the association between self-reported daily wellbeing entries and biometric data. Surprisingly, self-reported stress and nervousness had no association with resting HRV (Stressed HRV 63.5 ± 0.6 vs. Not Stressed HRV 63.1 ± 0.5 ms, *p* = 0.63) and (Nervous HRV 62.9 ± 0.9 vs. Not Nervous HRV 63.7 ± 0.4 ms, *p* = 0.41). Contrary to our hypothesis, self-reported feelings of being energized and motivated had a negative association with heart rate variability (Energized HRV 64.1 ± 0.4 vs. Not Energized 66.9 ± 0.8 ms, *p* < 0.01) and (Motivated HRV 63.1 ± 0.4 vs. Not Motivated HRV 65.3 ± 0.9 ms, *p* < 0.05).

Expectedly, feeling emotionally and mentally stable had a significant and positive association with HRV (Emotionally/Mentally Stable HRV 64.0 ± 0.4 vs. Not Emotionally/Mentally Stable HRV 60.7 ± 1.2 ms, *p* < 0.05). Naturally, alcohol consumption and feeling sick had a significant negative association with HRV (Alcohol HRV 54.5 ± 0.7 vs. No Alcohol 66.2 ± 0.6 ms, *p* < 0.001) and (Feeling sick HRV 59.3 ± 1.1 vs. Not Feeling Sick 61.9 ± 0.3 ms, *p* < 0.05). Hydrating sufficiently and feeling recovered had a significant and positive association with HRV (Hydrated Sufficiently HRV 65.4 ± 0.4 vs. Not Hydrated Sufficiently 59.6 ± 0.8 ms, *p* < 0.001) and (Recovered HRV 64.5 ± 0.5 vs. Not Recovered 61.7 ± 0.7 ms, *p* < 0.01). See Table 2 with all HRV and daily wellness survey questions. Subjects record a mean of 85 ± 12 days of HRV measurements, with 84% ± 23% of matched daily wellbeing entries showing great compliance and engagement. See Table 3 with the number of True and False entries for each wellness survey question and the percentage of those entries over 3 months across the group.

## 4. Discussion

Chronically suppressed HRV is a well-established predictor of stress, fatigue, and adverse health concerns [3,8]. Most commercially available wearable devices generate user guidance based on short-term fluctuations in HRV, typically utilizing 7- and 30-day averages. However, there is a paucity of research supporting the validity and usefulness of these recommendations.

The present study investigated whether daily HRV measurements from a commercially available wrist-worn activity tracker had an association with users’ self-reported wellbeing. Over a 3-month period, self-reported wellbeing questions and overnight HRV measurements were collected, demonstrating unique and relevant findings for those who develop and use wearable devices to guide their daily routines.

In the present study, associations between overnight HRV measurements and feelings of stress, nervousness, and being energized did not align with several scientific publications that have reported on the topic. The findings of the current study contrast with those reported by Hannon and colleagues, who analyzed 424 observations from self-reported wellbeing diaries collected over a 14-day period, with HRV measurements obtained each morning using a chest strap heart rate monitor. They noted higher values of HRV were credibly linked to more favorable self-reports of fatigue (β = 0.281, 95% HDI: 0.020 to 0.562), stress (β = 0.353, 95% HDI: 0.059 to 0.606), and sleep quality (β = 0.510, 95% HDI: 0.239 to 0.779) [3].

It is important to note that the literature states the parasympathetic nervous system predominates when someone is in a relaxed state, increasing HRV, while the sympathetic nervous system predominates in an arousal or energized state, decreasing HRV [13], which may explain the negative association that was observed between HRV measurementsand those who stated they “Felt Energized”. However, other publications have stated that the parasympathetic part of the autonomous nervous system may have a more predominant effect on HRV than the sympathetic system [14,15].

Another study explored the use of yoga to lower pre-test anxiety in undergraduate students. Thirteen undergraduate students (85% nursing majors, 15% awaiting program entry, 20  ±  4.9 years of age) completed a crossover study design, completing a yoga session or a controlled session (independent, quiet studying) the day before a test. Ratings of anxiety, salivary cortisol, and HRV measurements were collected. Yoga improved participants’ self-reported stress levels; however, these changes in perceived stress were not reflected in their HRV measurements [16].

Research indicates that training programs guided by HRV can meaningfully influence outcomes among highly trained endurance athletes [17]. Morinaga and Takai compared twelve highly trained runners divided into a predetermined aerobic training program and an HRV-guided aerobic training plan. The HRV-guided training group had large improvements in both maximal and submaximal aerobic power, despite undergoing a lower overall training load compared to those on the predetermined plan [18]. However, Medellín Ruiz and colleagues concluded in their systematic review and meta-analysis that HRV-based training did not provide a statistically significant benefit over predetermined training with highly trained endurance athletes [19].

Interestingly and aligned with the present results, Figueiredo and colleagues found that daily self-reported stress assessments could be leveraged to personalize endurance training prescriptions, resulting in improved performance. When combined with HRV metrics, these self-reports could contribute to a more comprehensive understanding of training-induced adaptations [20].

A few limitations need to be taken into consideration when interpreting these study results. The present study focused on the general population, whereas prior research often examined elite athletes or unhealthy groups, who typically show greater HRV suppression. Although HRV measured from PPG is a valid and reliable measurement at rest, it does not measure electrical activity directly. Instead, it infers heartbeat timing from blood flow changes, which may not be as precise as the gold-standard electrocardiogram method [1]. The sample size was small, lacked diversity, and skewed towards younger adults. Self-reporting bias may have occurred due to the binary nature of the questions, where subjects may have over- or under-reported their daily wellbeing status; a Likert scale would have been a better representation of individual wellbeing. Finally, due to the exploratory nature of this study, we did not conduct any multivariable modeling and tested each wellbeing entry independently, which can obscure associations and produce confounded findings. Future research should include more robust mixed-effects or GEE models to provide formal inferences for correlated observations that were displayed in this study. However, it is the opinion of the authors that this limitation does not take away from the overall observation that HRV readings may not always be associated with the subjective wellbeing of individuals and should be considered when making readiness scores and recommendations for wearable technologies.

## 5. Conclusions

In conclusion, the present study demonstrated that HRV biometrics alone did not reflect subjects’ feelings of nervousness, stress, or energy. The novelty of this study was the use of continuous monitoring of biometric data from a wearable activity tracker, conducted concurrently with daily self-reported wellbeing assessments over a three-month period. These outcomes demonstrate the need to elucidate the impact and use of HRV-guided recommendations versus or in tandem with self-reported measures of wellbeing.

## Figures and Tables

**Table 1:** Descriptive statistics and demographics. Mean ± SD.

		Males	Females
n		21	18
Age (years)		32 (7)	28 (4)
Height (cm)		182.6 (6.8)	168.3 (8.1)
Weight (kg)		92.4 (18.2)	71.8 (12.2)
BMI (kg/m^2^)		27.7 (4.0)	25.4 (4.0)
Race (n)	White	15	15
	Black or African American	1	0
	American Indian/Alaskan Native	1	0
	Asian	3	0
	Other	1	3
Ethnicity (n)	Non-Hispanic	19	15
	Hispanic	2	3

**Table 2:** Daily wellness survey questions vs. Whoop HRV measurements. Mean ± SE.

Daily Wellness Questions	True (HRV)	False (HRV)
Feel any Stress	63.5 (0.6)	63.1 (0.5)
Feel Emotionally/Mentally stable	64.0 (0.4) *	60.7 (1.2)
Feel Energized	64.1 (0.4) *	66.9 (0.8)
Feel Motivated	63.1 (0.4) *	65.3 (0.9)
Feel Sick or Ill	59.3 (1.1) *	61.9 (0.3)
Feel Nervous or Anxious	62.9 (0.9)	63.7 (0.4)
Felt Recovered	64.5 (0.5) *	61.7 (0.7)
Any Alcohol	54.5 (0.7) **	66.2 (0.6)
Any Caffeine	64.3 (0.4)	64.4 (1.0)
Hydrated Sufficiently	65.4 (0.4) **	59.6 (0.8)

* *p* < 0.05, ** *p* < 0.001.

**Table 3:** Daily wellness survey true and false responses. n (% of total responses).

Daily Wellness Questions	True	False	% of Completed Entries
Feel Any Stress	1089 (42.0%)	1506 (58.0%)	77.2%
Feel Emotionally/Mentally stable	2395 (89.6%)	277 (10.4%)	79.5%
Feel Energized	1942 (74.7%)	658 (25.3%)	77.4%
Feel Motivated	2134 (79.5%)	551 (20.5%)	79.9%
Feel Sick or Ill	264 (10.0%)	2389 (90.0%)	79.0%
Feel Nervous or Anxious	565 (21.0%)	2120 (79.0%)	79.9%
Felt Recovered	1879 (70.3%)	792 (72.0%)	79.5%
Any Alcohol	767 (28.0%)	1969 (0.6)	81.4%
Any Caffeine	2470 (86.0%)	403 (14.0%)	85.5%
Hydrated Sufficiently	2272 (82.3%)	490 (17.7%)	82.2%

## Data Availability

The raw data supporting the conclusions of this article will be made available by the authors upon request.

## Abbreviations

The following abbreviations are used in this manuscript:

HRV	Heart rate variability
RHR	Resting heart rate
PPG	Photoplethysmography
RMSSD	Root mean square of successive differences
